# Management of Type 1 Plastic Bronchitis—A Pediatric Case Report

**DOI:** 10.1002/ccr3.70406

**Published:** 2025-04-20

**Authors:** Aswathy Mathews, Amal Prazad

**Affiliations:** ^1^ Government Sivagangai Medical College Sivagangai Tamil Nadu India; ^2^ Memorial Health System Marietta Ohio USA

**Keywords:** bronchial casts, bronchoscopy, hypersecretion, plastic bronchitis

## Abstract

Plastic bronchitis (PB) is a rare and potentially fatal condition characterized by the formation of branching bronchial casts, leading to airway obstruction that can cause severe respiratory failure. We present the case of a 23‐month‐old male with a recent diagnosis of asthma who presented to our hospital with a worsening 7‐day fever and a 5‐day cough and shortness of breath. He had a history of two hospitalizations and multiple nebulizations with comparable symptoms before this appointment. His chest CT scan during his stay at our hospital revealed volume loss and consolidation with an air bronchogram in the lateral segment of the right middle lobe and the entire right lower lobe. Bronchoscopy showed that the bronchus intermedius was blocked by a bronchial mucus cast. After removal of the cast, the biopsy's histopathology revealed that the cast was made of fibrinous debris and inflammatory cells, predominantly eosinophils and a small number of neutrophils. As a result, this patient was given a working diagnosis of Type 1 plastic bronchitis. In treating this child's plastic bronchitis, our main objectives were to treat underlying problems, relieve acute airway obstructions, and stop further cast development. Bronchoalveolar culture revealed the growth of *Klebsiella pneumoniae* for which ceftazidime and avibactam were initiated. A follow‐up chest X‐ray showed a notable improvement. For both prevention and therapy, we started mucolytics and fibrinolytics for the patient. Montelukast, low‐dose azithromycin, bronchodilators, and inhaled corticosteroids were employed to treat the inflammation resulting from his plastic bronchitis. A metered dose inhaler containing budesonide (Budecort) was given to the patient upon discharge to reduce inflammation and enhance lower lung airflow. The patient was given urgent pediatric follow‐up on discharge to monitor symptom worsening/improvements. A high index of clinical suspicion is necessary for the diagnosis and management of plastic bronchitis (PB). Management entails ongoing medical care to address underlying diseases and avoid the need for additional casts, as well as the bronchoscopic removal of casts to relieve airway obstruction.

**Summary**

- Plastic bronchitis is a rare and potentially fatal condition requiring prompt diagnosis and comprehensive management to alleviate airway obstruction and prevent recurrence.
- Early intervention and ongoing care are critical for successful outcomes.

## 1 Introduction

Plastic bronchitis (PB) is a rare and potentially fatal condition characterized by the formation of branching bronchial casts, leading to airway obstruction and severe respiratory failure [1]. In this report, we present the case of a 23‐month‐old male recently diagnosed with asthma, who exhibited a worsening 7‐day fever, 5‐day cough, and shortness of breath upon hospital admission. The child's medical history included two previous hospitalizations and multiple nebulizations with similar symptoms. A chest CT scan revealed volume loss and consolidation with an air bronchogram in the lateral segment of the right middle lobe and the entire right lower lobe. Bronchoscopy identified a bronchial mucus cast obstructing the bronchus intermedius, and histopathology confirmed the cast comprising fibrinous debris and inflammatory cells, predominantly eosinophils, leading to a diagnosis of Type 1 PB. This case underscores the importance of early diagnosis and comprehensive management, including addressing underlying problems, relieving acute airway obstructions, and preventing further cast development, to improve patient outcomes.

## 2 Case Presentation

### 2.1 Case History and Examination

A 23‐month boy, the third child born by normal vaginal delivery (G3L3), had a 5‐day history of cough, a 7‐day history of fever, and a worsening respiratory dyspnea. For the last four months, these symptoms have been recurrent. The fever was described as intermittent, with a maximum temperature of 102°F and two to three daily spikes. The fever was not associated with rash, chills, or rigors. There were no diurnal fluctuations or post‐tussive vomiting symptoms, and the cough was nonproductive and nonspasmodic. There was no history of bottle feeding, oil instillation, cyanosis, suck–rest–suck cycle, greasy stools, failure to thrive, or Koch's contact. The birth was a full‐term normal vaginal delivery, 2.5 kg BCIAB (no birth trauma, no congenital abnormalities, no infections, no allergies, and no burns at birth), and no hospitalization in the neonatal intensive care unit. Developmental history was appropriate to age. According to the National Immunization Schedule, the immunization record was current and free of any unique immunizations. The dietary history covered 20 days of exclusive breastfeeding, as well as current cow's milk and supplemental feeding.

On physical examination, mild conjunctival pallor was noted; however, there was no clubbing, lymphadenopathy, or indications of vitamin insufficiency. Respiratory examination revealed that reduced air entry was detected by auscultation in the right posterior aspect, right‐sided infra‐axillary, and right‐sided inframammary region, with sporadic bilateral expiratory wheeze. A cardiovascular exam showed that S1 and S2 were present. There was no organomegaly, and the abdomen was soft and nontender without guarding. Neuro examination revealed normal reflexes, power, and tone in bilateral upper and lower extremities.

**FIGURE 1:**
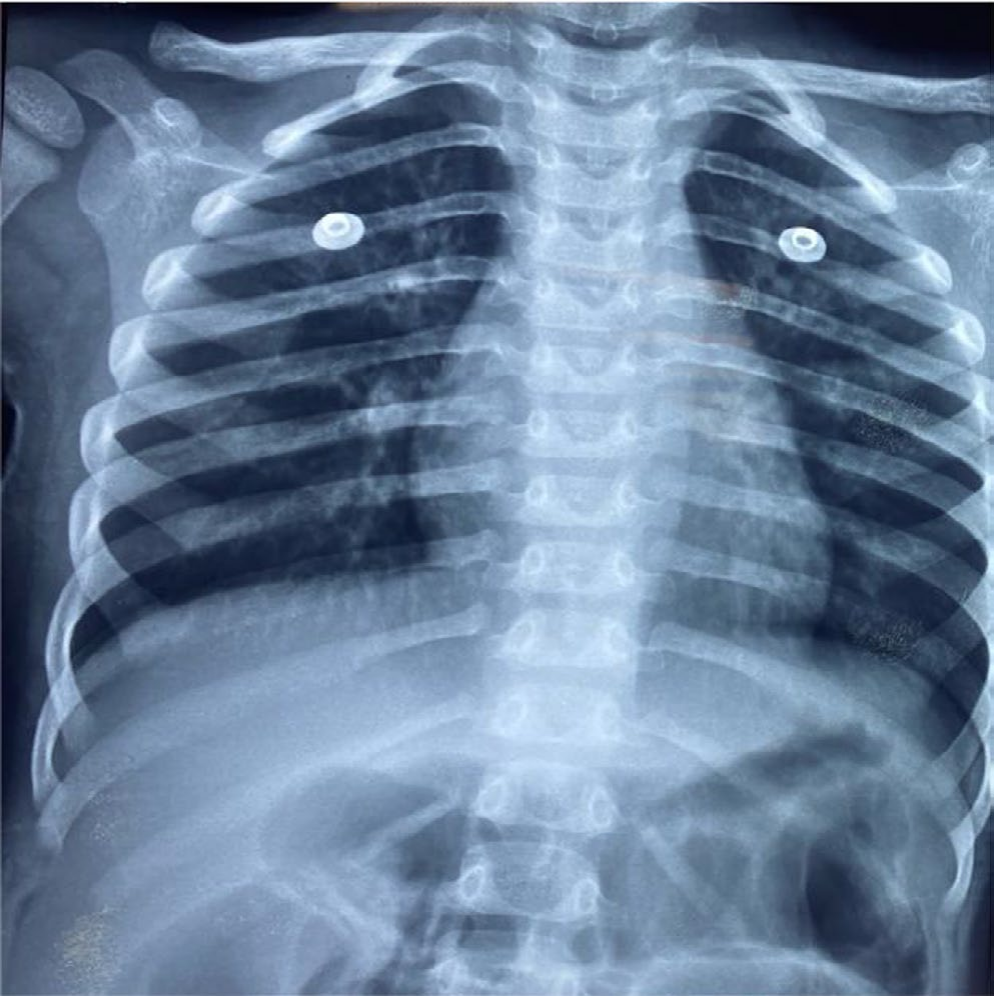
Chest X‐ray of first episode showing increased bronchoalveolar markings in the right lower zone.

The first episode of symptoms occurred in 3 months old, which included fever, cough, and increasing respiratory dyspnea. This event required a 4‐day hospital stay, including 1 day in the intensive pediatric care unit. Physical examination at this time revealed bilateral wheezing, tachypnea, intercostal and subcostal retractions, and crepitations. Increased bronchoalveolar marks in the right lower zone were visible on the chest X‐ray (Figure 1). Lab work showed a hemoglobin count of 9.9, white blood cells of 13,000 and platelets of 400,000. The working diagnosis at that time was community‐acquired pneumonia of the right lower lobe. After receiving nebulizations of salbutamol and budesonide, oral fluticasone, and intravenous antibiotics (Monocef), the youngster was released with pediatric follow‐up.

**FIGURE 2:**
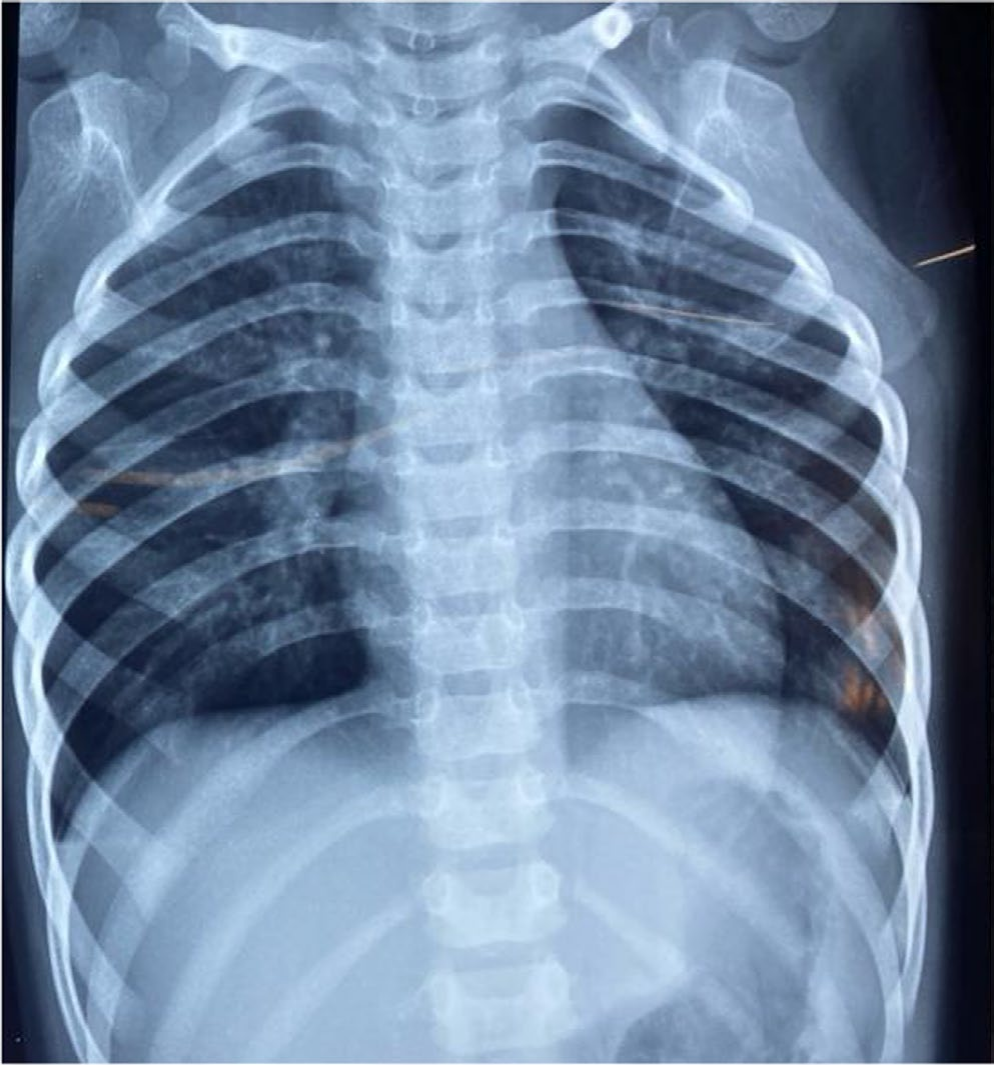
Chest X‐ray of second episode showing increased bronchoalveolar.

The second episode happened about 3 months ago from the latest presentation. This time, the child had a 1‐day history of coughing and worsening respiratory distress, which required a 3‐day stay in the ward. The physical examination was comparable to those of the first episode. Increased bronchoalveolar marks in the right lower zone were once again visible on the chest X‐ray (Figure 2). Hemoglobin level of 8.5, white blood count of 14,000, platelet 300,000, and C‐reactive protein 58.2 mg/L were the results of the complete blood count. One dosage of magnesium sulfate, augmentin, and nebulizations with budesonide and albuterol/ipratropium was part of the treatment. Upon discharge, the youngster was started on a metered dose of salbutamol and budesonide.

**FIGURE 3:**
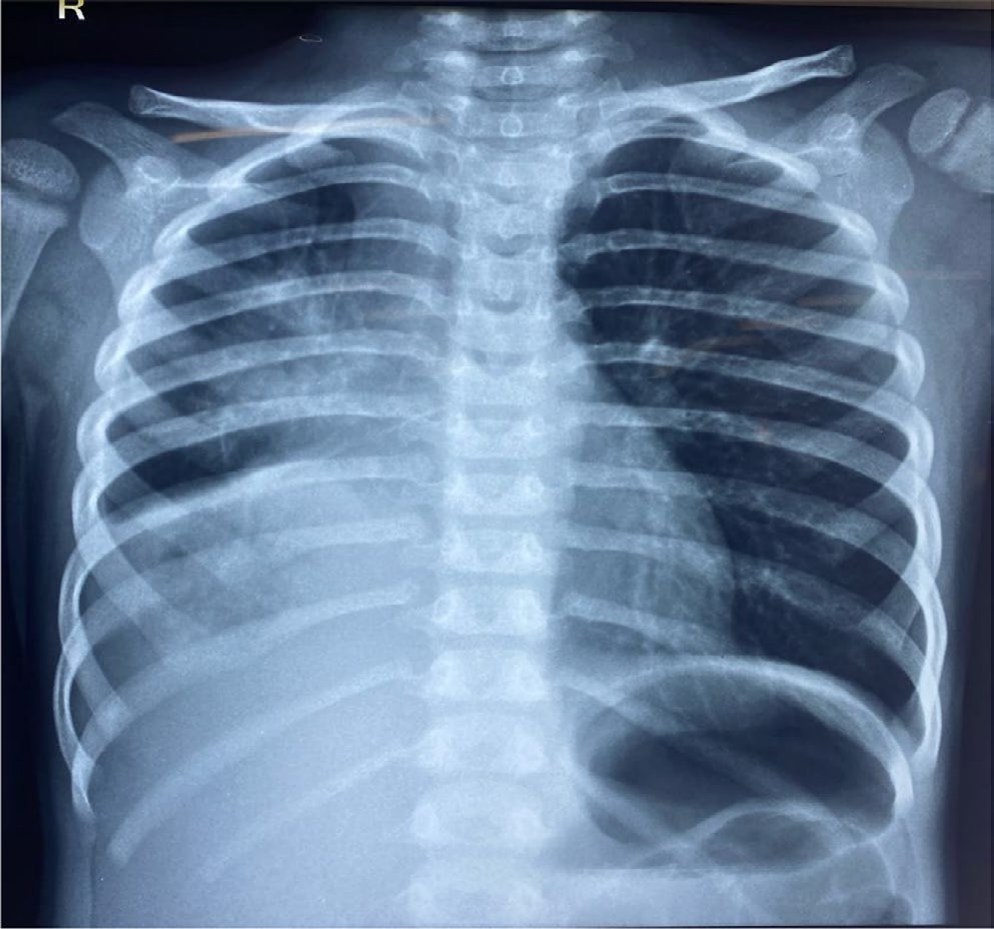
Chest X‐ray from the latest episode showing consolidation and collapse of the right lower lobe.

During the latest episode, the child presented with a worsening 7‐day fever and a 5‐day cough and shortness of breath. Vitals on presentation included a heart rate of 112/min, respiratory rate of 32/min, blood pressure 96/60 mmHg, and spO_2_ 94% on 2 L supplemental O_2_. Lab work was significant for anemia and leukocytosis (Table 1, Table 2, and Table 3). Tachypnea, subcostal and intercostal retractions, reduced air entry on the right side, bilateral crepitations, and bilateral wheezing were evident on pulmonary examination. Other physical examination findings were insignificant. An X‐ray of the chest revealed consolidation and collapse of the right lower lobe (Figure 3). Methicillin‐resistant *Staphylococcus aureus* was found in his blood culture, but the tuberculosis workup came out negative. The patient immediately received nebulizations of salmeterol and budesonide, as well as injections of meropenem, vancomycin, magnesium sulfate, and aminophylline.

**TABLE 1:** Complete blood count—significant values.

Parameter	Value
HB	10.7
MCV/MCH/RDW	68.2/20/21.1
WBC	13,000 (N‐ 28 L‐ 51 M‐6.8 E‐ 13.5)
PLT	380,000

Abbreviations: E, eosinophils; HB, hemoglobin; L, lymphocytes; M, monocytes; MCH, mean corpuscular hemoglobin; MCV, mean corpuscular volume; N, neutrophils; PLT, platelets; RDW, red cell distribution width; WBC, white blood cell count.

**TABLE 2:** Comprehensive metabolic panel—significant values.

Parameter	Value
BUN/Creat	13/0.23
SGOT/SGPT	25/11
Na/K	139/4.7

Abbreviations: BUN, blood urea nitrogen; Creat, creatinine; K, potassium; Na, sodium; SGOT (AST), serum glutamic oxaloacetic transaminase (aspartate aminotransferase); SGPT (ALT), serum glutamic pyruvic transaminase (alanine aminotransferase).

**TABLE 3:** Inflammatory values.

Parameter	Value
ESR	6
Adeno PCR	Negative
CRP	0.7

Abbreviations: Adeno PCR, adenovirus polymerase chain reaction; CRP, C‐reactive protein; ESR, erythrocyte sedimentation rate.

### 2.2 Workup/Treatment

**FIGURE 4:**
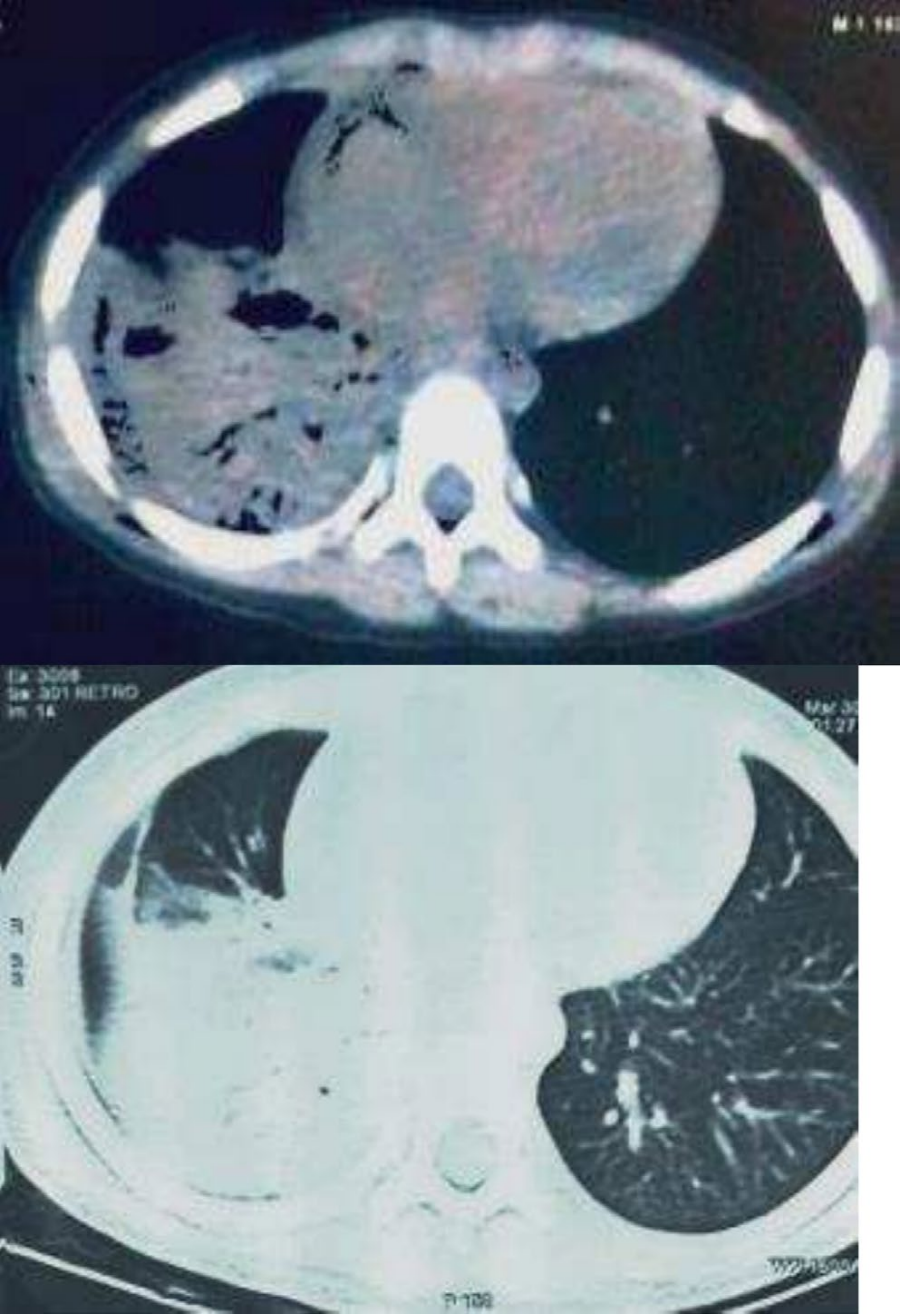
HRCT scan of the chest showing lateral sections of the right middle lobe and the entire right lower lobe showed consolidation with air bronchogram, which was linked to volume loss.

**FIGURE 5:**
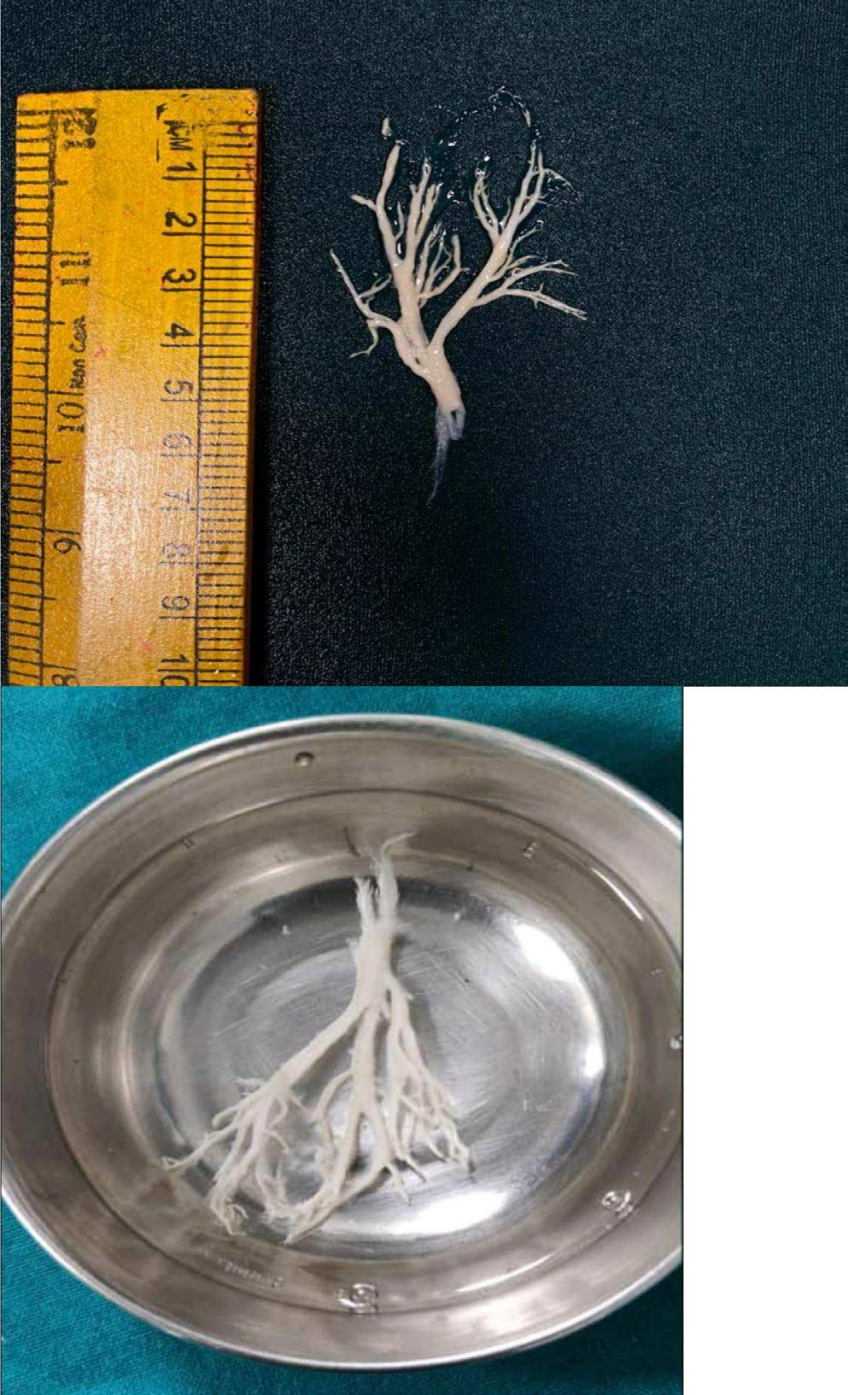
Bronchial cast after bronchoscopy removal.

CT scan of the chest was performed due to recurrent episodes of pneumonia and consolidation abnormalities in the chest X‐ray. The lateral sections of the right middle lobe and the entire right lower lobe showed consolidation with air bronchogram, which was linked to volume loss (Figure 4). A repeat chest radiograph showed bronchiectasis or collapse of the affected lung region distal to the location of the chronic blockage. Bronchoscopy was performed with informed consent from the patients, which revealed viewing of a bronchial cast. The entire cast was removed during the procedure, and a biopsy was sent for histopathological evaluation (Figure 5). The histopathology of bronchial casts revealed numerous inflammatory cells and fibrinous materials. Eosinophils made up most of the inflammatory cells, with neutrophils making up a significantly smaller portion. Bronchoalveolar lavage showed 15% of eosinophils. *Klebsiella pneumoniae*, which was also detected by bronchoalveolar lavage culture, was treated with ceftazidime and avibactam.

### 2.3 Differential Diagnosis

The differential diagnosis for this case includes bronchiectasis, asthma, foreign body aspiration, chronic bronchitis, congenital heart disease, lymphatic abnormalities, and infections. To rule out these conditions, a thorough clinical history, physical examination, and appropriate investigations were conducted. Bronchiectasis was considered due to recurrent pneumonia and persistent cough, but the absence of chronic productive cough and the presence of bronchial casts on bronchoscopy favored PB. Asthma was ruled out due to the lack of a family history, the episodic nature of symptoms, and the presence of bronchial casts. Foreign body aspiration was excluded based on the absence of a witnessed choking event and the presence of bronchial casts on bronchoscopy. Chronic bronchitis was unlikely due to the patient's age and lack of exposure to irritants. Congenital heart disease was ruled out based on a normal cardiovascular examination and the absence of cyanosis. Lymphatic abnormalities were considered but ruled out based on the absence of edema and other systemic signs. Finally, infections were ruled out based on a negative tuberculosis workup and the presence of bronchial casts on bronchoscopy.

### 2.4 Outcome/Follow‐Up

**FIGURE 6:**
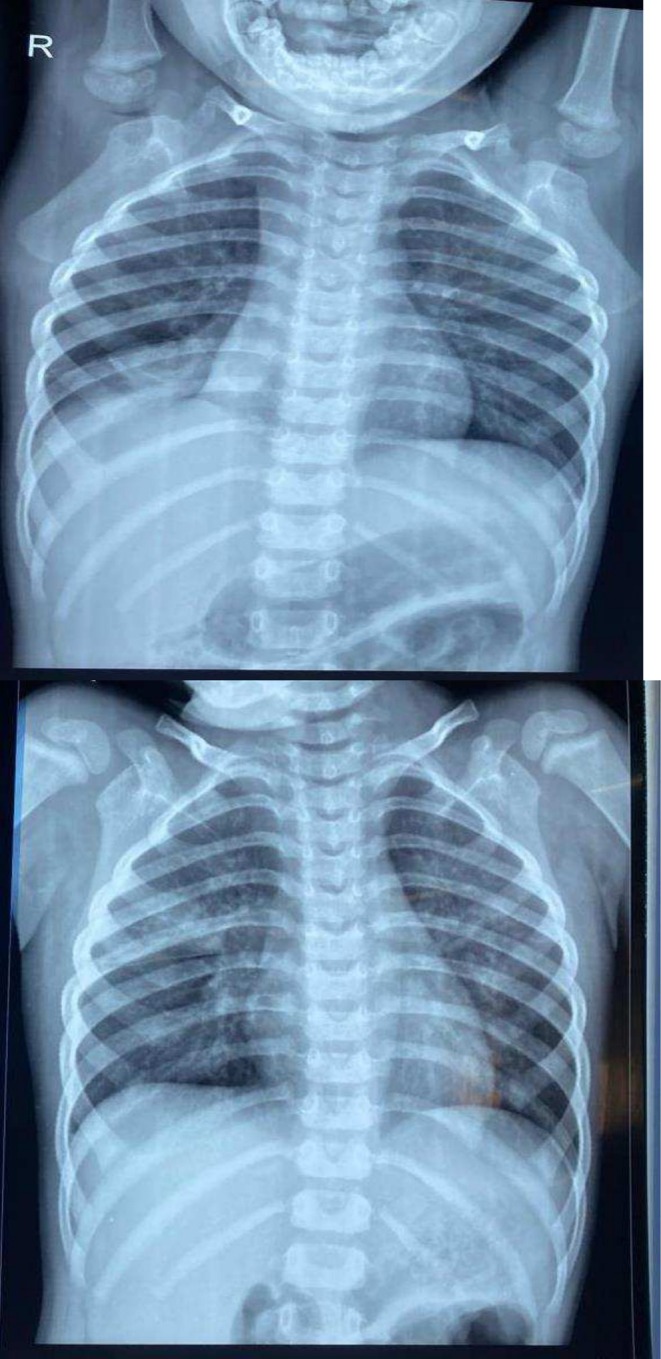
Follow‐up chest X‐ray revealing fewer evidence of opacification and clearer lung fields.

The patient was discharged on MDI budesonide with frequent follow‐ups with our pediatrician. A follow‐up chest X‐ray revealed reduced opacification and clearer lung fields (Figure 6). The patient was prescribed salbutamol, fluticasone, montelukast, and dornase alfa (Pulmozyme) during the urgent follow‐up that preceded this presentation. Moreover, the patient was prescribed N‐acetylcysteine (Mucomyst) and aerosolized heparin (Hepalin) by the pediatrician to prevent recurrence. To reduce airway inflammation, leukotriene inhibitors (montelukast), low‐dose azithromycin for 10 days, inhaled corticosteroids (fluticasone), and bronchodilators (salbutamol and ipratropium bromide) were provided.

## 3 Discussion

Branching cohesive casts can induce partial or complete airway blockage in PB, a rare disease. Since its first description in 1902, PB has been classified into various categories based on its etiology and anatomical location. These categories include idiopathic, inflammatory, infectious, allergic, malignancy‐associated, and congenital heart disease–related forms [2]. There are two forms of PB: Type 1, which has inflammatory casts that are highly cellular and frequently related to disorders like cystic fibrosis and asthma (such as seen in the presented patient), and Type 2, which has acellular casts that are linked to the postsurgical correction of congenital heart problems [3]. The case noted here would be categorized as Type 1 PB as the patient had a history of asthma and had histologic evidence of inflammatory mediators, particularly eosinophils.

Cast histological examination may reveal underlying conditions and help guide treatment choices. Inhaled and systemic corticosteroids, N‐acetylcysteine, and long‐term low‐dose macrolide antibiotics (which have anti‐inflammatory and immunomodulatory qualities as well as mucolytic activities that help avoid exacerbations) are used to reduce bronchial cast development [4]. PB typically manifests as a side effect of underlying illnesses, which can impact the patient's prognosis. Asthma, cystic fibrosis, sickle cell disease, lymph vessel abnormalities, and congenital cardiopathies are among the conditions frequently linked to PB [5]. Studies have shown that patients with underlying asthma or allergic diseases who develop PB have a mortality rate of 16%, while those with underlying heart abnormalities have a mortality rate of 29% [6].

Both pharmaceutical and procedural methods are used to treat PB, and bronchoscopy is crucial for both diagnosis and treatment. For the treatment of PB and its refractory form, bronchoscopic removal of the cast and/or cryotherapy are safe and efficient procedures [7]. The best technique for mechanical cast disruption is flexible or rigid bronchoscopy, which is frequently assisted by contrast‐enhanced CT imaging [8]. According to research studies, repeated rigid bronchoscopies are frequently required for the successful treatment of recurrent PB [5, 9]. Cast mobilization is also aided by chest physical therapy. Heparin inhalation should be considered when the casts are mostly fibrin, and the underlying illnesses are not well addressed [10]. To lessen bronchial cast formation and symptoms, long‐term low‐dose macrolide antibiotics (which have anti‐inflammatory and immunomodulatory properties as well as mucolytic actions that assist prevent exacerbations), inhaled and systemic corticosteroids, and N‐acetylcysteine are utilized [4]. Biologic agents such as omalizumab have shown promise in preventing the recurrence of PB, especially in those with elevated IgE levels [11].

## 4 Conclusion

PB is a rare and complicated disorder that can manifest in both pediatric and adult patients. Here, we presented a pediatric patient with a history of asthma presenting with cough and shortness of breath who was diagnosed with PB. This patient was successfully managed with timely interventions such as HRCT of the chest and bronchoscopy with biopsy. Furthermore, leukotriene inhibitors (montelukast), low‐dose azithromycin for 10 days, inhaled corticosteroids (fluticasone), bronchodilators (salbutamol and ipratropium bromide), N‐acetylcysteine (Mucomyst), and aerosolized heparin (Hepalin) were also provided for the management of this disease.

This case report illustrates the diagnostic workup and management of PB. Diagnosis of PB involves a combination of clinical symptoms, laboratory findings, and imaging findings, reinforcing the importance of a comprehensive evaluation. Although a good prognosis is common for most patients, recurrence of the disease is noted without proper preventive management. As our understanding of PB evolves, so too will our ability to provide targeted and effective treatments that improve outcomes for individuals affected by this disorder.

## Author Contributions

**Aswathy Mathews:** data curation, formal analysis, investigation, project administration, supervision, writing – original draft, writing – review and editing. **Amal Prazad:** conceptualization, formal analysis, project administration, validation, writing – original draft, writing – review and editing.

## Ethics Statement

No formal ethical approval was required for this case report. All identifying details were de‐identified to maintain confidentiality.

## Consent

Written informed consent for publication of this case report, including associated images and data, was obtained from the patient's parents as the patient is a minor, in accordance with institutional guidelines.

## Conflicts of Interest

The authors declare no conflicts of interest.

## Data Availability

Data sharing is not applicable to this case report as all relevant information, including images and supporting details, is comprehensively presented within the manuscript. For any additional information or inquiries, please contact the corresponding author.
